# Insulinotropic Effect of the Non-Steroidal Compound STX in Pancreatic β-Cells

**DOI:** 10.1371/journal.pone.0034650

**Published:** 2012-04-10

**Authors:** Ana B. Ropero, Paloma Alonso-Magdalena, Sergi Soriano, Pablo Juan-Picó, Troy A. Roepke, Martin J. Kelly, Ángel Nadal

**Affiliations:** 1 Instituto de Bioingeniería and CIBER de Diabetes y Enfermedades Metabólicas Asociadas (CIBERDEM), Universidad Miguel Hernández, Elche, Spain; 2 Department of Physiology and Pharmacology, Oregon Health & Science University, Portland, Oregon, United States of America; University of Córdoba, Spain

## Abstract

The non-steroidal compound STX modulates the hypothalamic control of core body temperature and energy homeostasis. The aim of this work was to study the potential effects of STX on pancreatic β-cell function. 1–10 nM STX produced an increase in glucose-induced insulin secretion in isolated islets from male mice, whereas it had no effect in islets from female mice. This insulinotropic effect of STX was abolished by the anti-estrogen ICI 182,780. STX increased intracellular calcium entry in both whole islets and isolated β-cells, and closed the K_ATP_ channel, suggesting a direct effect on β-cells. When intraperitoneal glucose tolerance test was performed, a single dose of 100 µg/kg body weight STX improved glucose sensitivity in males, yet it had a slight effect on females. In agreement with the effect on isolated islets, 100 µg/kg dose of STX enhanced the plasma insulin increase in response to a glucose load, while it did not in females. Long-term treatment (100 µg/kg, 6 days) of male mice with STX did not alter body weight, fasting glucose, glucose sensitivity or islet insulin content. Ovariectomized females were insensitive to STX (100 µg/kg), after either an acute administration or a 6-day treatment. This long-term treatment was also ineffective in a mouse model of mild diabetes. Therefore, STX appears to have a gender-specific effect on blood glucose homeostasis, which is only manifested after an acute administration. The insulinotropic effect of STX in pancreatic β-cells is mediated by the closure of the K_ATP_ channel and the increase in intracellular calcium concentration. The *in vivo* improvement in glucose tolerance appears to be mostly due to the enhancement of insulin secretion from β-cells.

## Introduction

The islet of Langerhans is a key tissue involved in maintaining blood glucose homeostasis and its dysfunction is an essential factor in the development of type 1 and type 2 Diabetes Mellitus [1], [2]. In pancreatic β-cells, ATP-sensitive potassium channels, K_ATP_, play a crucial role in glucose-stimulated insulin secretion. At low glucose concentrations, K_ATP_ channels are open and the resting potential of pancreatic β-cells remains close to −70 mV. When blood glucose levels increase, [ATP]/[ADP] ratio rises, closing the K_ATP_ channel. As a consequence, membrane potential depolarizes up to about −40 mV, which opens voltage-dependent calcium channels, induces Ca^2+^ influx and in turn activates insulin secretion. This follows a pulsatile pattern as a consequence of the oscillatory pattern in intracellular calcium concentration ([Ca^2+^]_i_) [3]–[5].

Estrogens are known to be important regulators of blood glucose homeostasis through their action on the different tissues involved in maintaining glycemia, including the islets of Langerhans [6]–[8]. In fact, different situations characterized by a deficiency in estrogenic activity are associated with glucose intolerance and insulin resistance [9]–[12]. In addition, two important epidemiological studies show the reduced incidence of Diabetes in postmenopausal women following a combined estrogen-progestin hormonal therapy [13], [14]. However, the severe side effects of this hormonal replacement therapy, such as increased senile dementia, ovarian cancer and ischemic stroke makes the use of estrogens as therapeutic anti-diabetic drugs complicated [15]–[17].

Estrogens modulate pancreatic β-cell function through both ERα and ERβ. Physiological concentrations of 17β-estradiol exert insulinotropic effects through ERβ, while they increase insulin biosynthesis through ERα [7], [18], [19]. The involvement of a yet unknown receptor in the rapid insulinotropic effect of 17β-estradiol has not been ruled out [19], [20]. GPR30 may be involved in the modulation of pancreatic β-cell function and survival but only at supraphysiological concentrations of 17β-estradiol [21]–[23] (for discussion see [24]).

STX is a non-steroidal diphenylacrylamide compound structurally related to 4-hydroxytamoxifen [25], [26]. STX has some estrogenic properties, including the prevention of body weight gain and the reduction in body core temperature induced by ovariectomy in guinea pigs. In addition, STX modulates the activity of hypothalamic POMC and dopamine neurons and increases K_ATP_ channel activity in hypothalamic GnRH neurons [25], [27]–[30]. It also protects against ischemia-induced hippocampal neuron loss [31]. The effects of STX in hypothalamic neurons are inhibited by the specific antiestrogen ICI 182,780 [27], [30]. However, STX does not bind to ERα or ERβ nor does it have uterotropic effects [25]. Instead, it has been suggested to act through a new receptor determined to be a Gq-coupled mER [32]. Some of the actions of 17β-estradiol in hypothalamic POMC and dopamine neurons have been also proposed to be mediated through this Gq-coupled mER [32].

Therefore, the aim of this work is to test whether STX has estrogenic-like effects in pancreatic β-cells and whether these effects may be potentially useful from a therapeutic point of view.

## Materials and Methods

### Ethics Statement

The committee on internal animal care and use of the Miguel Hernández University reviewed and approved all the procedures followed (approval ID number: IB-ARL-001-10).

### Materials

Fura-2AM was obtained from Molecular Probes (Invitrogen, Barcelona). ICI 182,780 was from Tocris Cookson Ltd (Avonmouth, United Kingdom). STX was synthesized by a contract research organization under the direction of Drs. Tom Scanlan and Martin Kelly at the Oregon Health & Science University. All other chemicals were obtained from Sigma (Madrid).

### Animals

Gonadal intact adult male and female OF1 and male C57/BL6 mice were used aged 3–4months old. Females were ovariectomized using Isoflurane (5% for induction; 1–2% for maintenance). They were allowed to recover for at least 14 days before performing experiments. A model for mild diabetes was used by injecting (i.p.) C57/BL6 mice with 1 g/Kg nicotinamide and 30 min later with 150 mg/Kg streptozotocin (STZ). Nicotinamide partially protects pancreatic β-cells against the selective cytotoxic action of STZ. This produces a model of moderate hyperglycemia combined with the loss of early-phase insulin secretion [33]–[35]. Ten days after this treatment, surviving mice were used for *in vivo* experiments. All animals were kept under standard housing conditions.

### Islet and islet cells isolation

Pancreatic islets of Langerhans were isolated by collagenase (Sigma, Madrid, Spain) digestion as previously described [36]. Freshly isolated islets were used for calcium and insulin secretion measurements after 2 h recovery. For experiments using isolated β-cells, islets were dispersed into single cells with trypsin. Cells were then centrifuged and resuspended in RPMI 1640 without phenol-red (Invitrogen, Barcelona) and with 10% charcoal dextran-treated fetal bovine serum (Hyclone, USA), 2 mM L-glutamine, 200 U/ml penicillin and 0.2 mg/ml streptomycin. Cells were then plated in covers and used within 24 hours of initiating the culture.

### Recording [Ca^2+^]_i_

Freshly isolated islets of Langerhans or isolated islet cells were loaded with 5 µM Fura-2 AM for at least 1 hour at room temperature. Calcium recordings in both islets and isolated cells were obtained by imaging intracellular calcium under an inverted epifluorescence microscope (Zeiss, Axiovert 200). Images were acquired every 2 s with an extended Hamamatsu Digital Camera C4742-95 (Hamamatsu Photonics, Barcelona, Spain) using a dual filter wheel (Sutter Instrument CO, CA, USA) equipped with 340 nm and 380 nm, 10 nm bandpass filters (Omega optics, Madrid, Spain). Data were acquired using Aquacosmos software from Hamamatsu (Hamamatsu Photonics, Barcelona, Spain). Fluorescence changes are expressed as the ratio of fluorescence at 340 nm and 380 nm (F_340_/F_380_). Results were plotted and analyzed using commercially available software (Sigmaplot, Jandel Scientific). Area under the curve (AUC) was measured before STX application and during STX application after discarding the first minute of stimulus. The time interval was 10 min for islets and 15 min for isolated cells.

### Insulin secretion measurements

Freshly isolated islets were left to recover in the isolation medium for 2 hours in the incubator. After recovery, groups of 5 were transferred to 400 µl of a buffer solution containing 140 mM NaCl, 4.5 mM KCl, 2.5 mM CaCl_2_, 1 mM MgCl_2_, 20 mM HEPES and the corresponding glucose concentration with final pH at 7.4. The islets were kept for 1 hour in the incubator. Afterwards, 100 µl of the corresponding buffer solution with 5% BSA was added, incubated at room temperature for 3 min and let to cool down for 15 min on ice. Then, the medium was collected and insulin was measured in duplicate samples by radioimmunoassay using a Coat-a-Count kit (Siemens, Los Angeles, CA, USA). Insulin secretion was expressed as (μUI/islet×h). Increasing concentrations of glucose were used as internal controls. Fasting glucose levels were used to test the compound (7–8 mM for mice: [37]).

### Patch-clamp recordings

K_ATP_ channel activity was recorded using standard patch clamp recording procedures from isolated pancreatic β-cells cells. Currents were recorded using an Axopatch 200B patch-clamp amplifier (Axon Instruments Co. CA, USA). Patch pipettes were pulled from borosilicate capillaries (Sutter Instruments Co. CA, USA) using a Flaming/Brown P-97 micropipette puller (Sutter Instruments Co. CA, USA) with resistance between 3–5 MΩ when filled with the pipette solution as specified below. Bath solution contained (in mM): 5 KCl, 135 NaCl, 2.5 CaCl_2_, 10 Hepes and 1.1 MgCl_2_ (pH 7.4) and supplemented with glucose as indicated. The pipette solution contained (in mM): 140 KCl, 1 MgCl_2_, 10 Hepes and 1 EGTA (pH 7.2). The pipette potential was held at 0 mV throughout recording. K_ATP_ channel activity was quantified by digitising 60 s sections of the current record, which was filtered at 1 kHz and sampled at 10 kHz by a Digidata 1322A (Axon Instruments Co. CA, USA), and calculating the mean open probability of the channel (*NP_o_*) during the sweep. Channel activity was defined as the product of *N*, the number of functional channels, and *P_o_*, the open-state probability. *P_o_* was determined by dividing the total time channels spent in the open state by the total sample time. Data sampling was started 1 min before (control) and 5 min after application of test substances. Experiments were carried out at room temperature (20–24°C).

### Short-term treatment with STX

Mice were placed in separate cages the evening before the experiment. A single dose of 100 µg STX/kg body weight was inoculated in a volume of 150 µl saline solution just before the corresponding tests. For the Intraperitoneal Glucose Tolerance Test (IPGTT) mice fasted for 15 hours and 2 g glucose/kg body weight was injected. The Insulin Tolerance Test (ITT) was performed in the morning at 10 am and a dose of 1 UI insulin/kg body weight was used. All the injections were intraperitoneal. Blood glucose was obtained from the tail vein using an Accu-check portable glucometer (Roche Diagnostic GmbH, Mannheim, Germany). For glucose sensitivity, we quantified the glycemic response measuring the incremental area under the curve (AUC) [38], [39]. In a different set of experiments, plasma insulin was quantified 30 min after the glucose load as for the IPGTT. For this, mice were anesthetized with pentobarbital and blood was obtained by cardiac puncture with a syringe containing EDTA. Levels of plasma insulin were determined by ELISA using the ultrasensitive mouse insulin assay kit from Mercodia AB (Uppsala, Sweden).

### Long-term treatment with STX

Mice were injected intraperitoneally in the morning with a daily dose of 100 µg STX/kg body weight for 6 days. On the evening of the 6^th^ day, body weight was measured. On the morning of the 7^th^ day, an IPGTT was performed as described previously and islets were isolated from those mice. These islets were lysed in an ethanol/HCl buffer immediately after isolation. They were kept overnight at 4°C, the supernatant was collected by centrifugation the following day and the insulin content was measured using RIA.

### Statistical analysis

Data are expressed as mean ± SEM. Student's t-test was used for statistical comparison, unless otherwise stated. A probability level of 0.05 or lower was considered statistically significant.

## Results

### STX enhances glucose-induced insulin secretion in islets from male mice in an ICI 182,780-dependent manner

**Figure 1:**
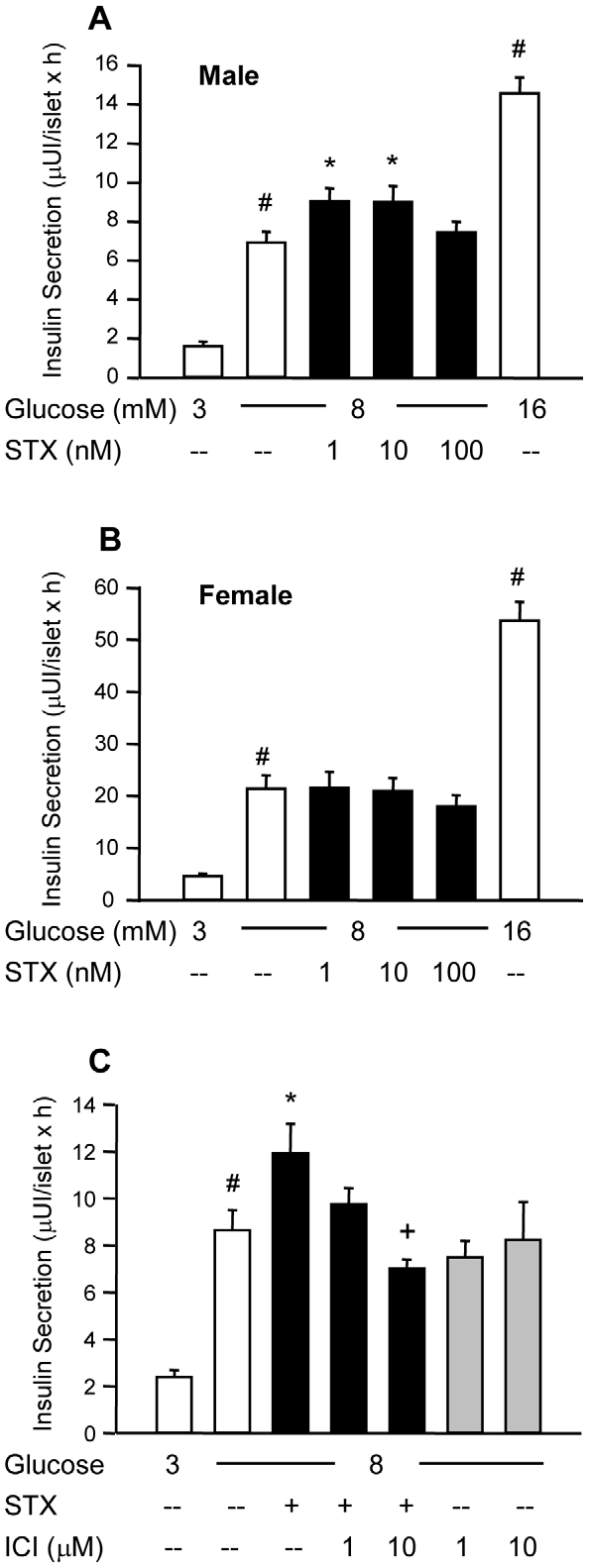
STX enhances glucose-induced insulin secretion in islets from male mice following a dose-response curve. A, Measurement of insulin secretion in isolated islets from male mice at different STX concentrations in the presence of 8 mM glucose. The insulin secretion in response to 16 mM glucose was used as an internal control. Insulin secretion experiments were performed for 1 hour, in groups of 5 islets. At least 75 islets were used per condition, from 12 different mice. B, The same as in A, but with islets from female mice. At least 110 islets were used per condition in groups of 5, from 15 different mice. C, Insulin secretion in response to 8 mM glucose in the absence or presence of 10 nM STX, 1 µM or 10 µM ICI 182,780 as indicated. Between 20 and 80 islets were used per condition in groups of 5, from 9 different mice. * p<0.05 *vs.* 8 mM glucose; # p<10^−4^ *vs.* 3 mM glucose; +p<0.05 *vs.* STX alone.

We first determined whether STX was capable of modulating pancreatic β-cell function. For this purpose, we performed separate insulin secretion experiments for islets from male and female mice. We observed that 1–10 nM STX produced an increase in glucose-induced insulin secretion in isolated islets from male mice by 24% and 31%, respectively, while 100 nM STX had no effect (Fig. 1A). However, this potentiating effect of STX was not obtained in islets from intact female mice (Fig. 1B). The specific anti-estrogen ICI 182,780 partially inhibited the insulinotropic effect of STX at 1 µM, while it completely blocked STX effects at 10 µM (Fig. 1C). Therefore, low concentrations of STX enhance glucose-induced insulin secretion in islets from male mice in an ICI 182,780-dependent manner.

### STX increases calcium entry into β-cells

**Figure 2:**
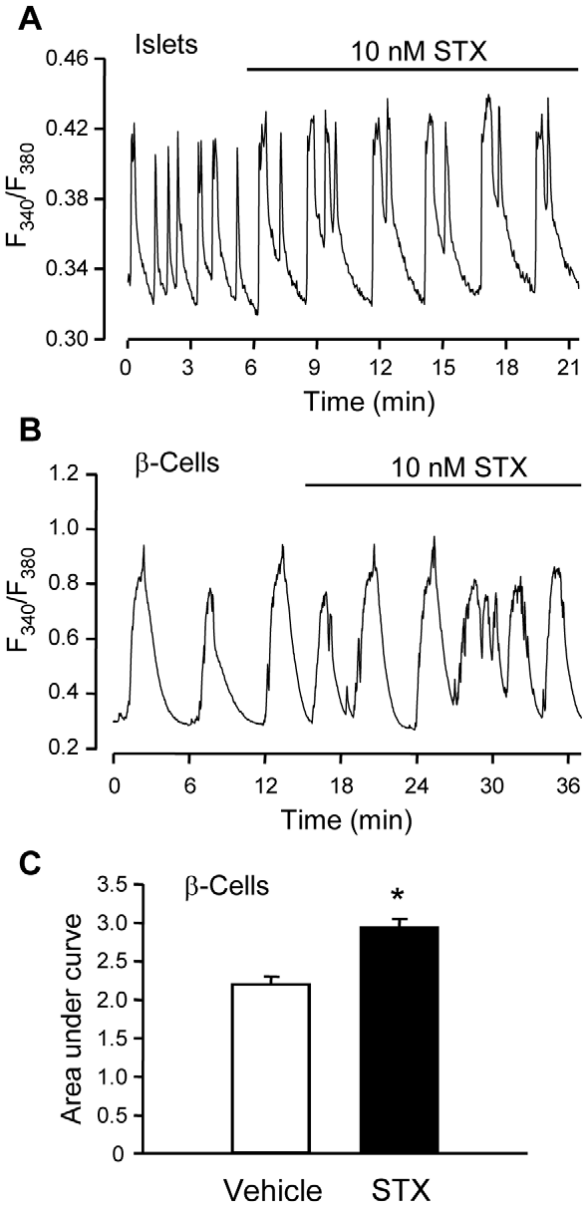
STX enhances calcium entry in islets and isolated β-cells. A, [Ca^2+^]_i_ recording of an islet of Langerhans in the presence of 8 mM glucose; 10 nM STX was added to the perfusion when indicated; n = 11 islets from 3 different male mice. B, [Ca^2+^]_i_ recording of an isolated pancreatic β-cell in the presence of 8 mM glucose; 10 nM STX was added to the perfusion when indicated. C, Area under the curve in [Ca^2+^]_i_ recordings from isolated β-cells. * p<0.000005; n = 100 cells from 3 different male mice.

Since insulin secretion is a calcium-driven event, we studied the effects of STX on [Ca^2+^]_i_ in isolated islets of Langerhans from male mice. 10 nM STX altered [Ca^2+^]_i_ in response to 8 mM glucose (Fig. 2A). The total calcium entry into the islets in response to 8 mM glucose in the absence or presence of 10 nM STX was quantified as the area under the curve (AUC). STX increased AUC in islets of Langerhans by 24%. We also recorded [Ca^2+^]_i_ in isolated β-cells (Fig. 2B). Similar to the effect in whole islets, STX increased calcium entry in the presence of 8 mM glucose in isolated β-cells (Fig. 2B, C). Therefore, 10 nM STX modulates intracellular calcium concentrations by increasing calcium influx into pancreatic β-cells.

### STX modulates the K_ATP_ channel activity in pancreatic β-cells

**Figure 3:**
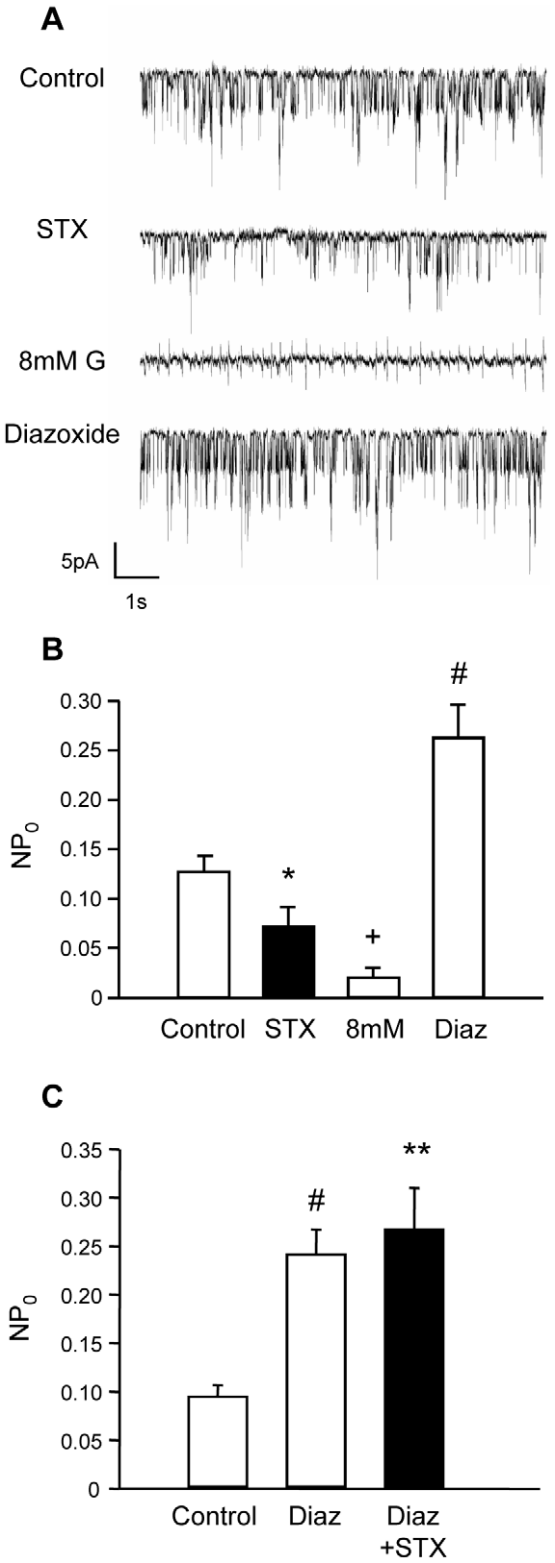
STX decreases the K_ATP_ channel activity. A, A cell-attached patch-clamp recording of a representative isolated β-cell is shown. Several seconds of the same recording are shown at different moments of the experiment. B, The open probability (NP_0_) of the K_ATP_ channel during Control (0 mM glucose), 10 nM STX, 8 mM glucose and 100 µM Diazoxide (Diaz) was measured. C, The open probability (NP_0_) of the K_ATP_ channel during Control (0 mM glucose), 100 µM Diazoxide (Diaz) and Diazoxide plus 10 nM STX (Diaz+STX). * p<0.05, ** p<0.01, # p<0.005, +p<0.00005 *vs.* Control; n = 11 cells from 5 different male mice.

As mentioned previously, K_ATP_ channels are key to regulating glucose-induced insulin secretion. Therefore, we decided to study whether the insulinotropic effect of STX in islets from male mice was mediated by the regulation of the channel activity. For this purpose, cell-attached recordings of isolated β-cells were performed in the absence of glucose (Control, Fig. 3A). The addition of 10 nM STX produced a significant decrease in the activity of the K_ATP_ channel (Fig. 3A–B). This decreased represented 43% of the activity of the channel in the absence of glucose (Control). Channels were perfectly functional after washout of STX, since they responded to 8 mM glucose and to the K_ATP_ opener diazoxide as expected (Fig. 3A–B). In addition, diazoxide prevented the decrease in activity elicited by STX as shown in figure 3C. Therefore, 10 nM STX modulates the activity of the K_ATP_ channel in pancreatic β-cells.

### *In vivo* administration of STX improves glucose tolerance in male mice

**Figure 4:**
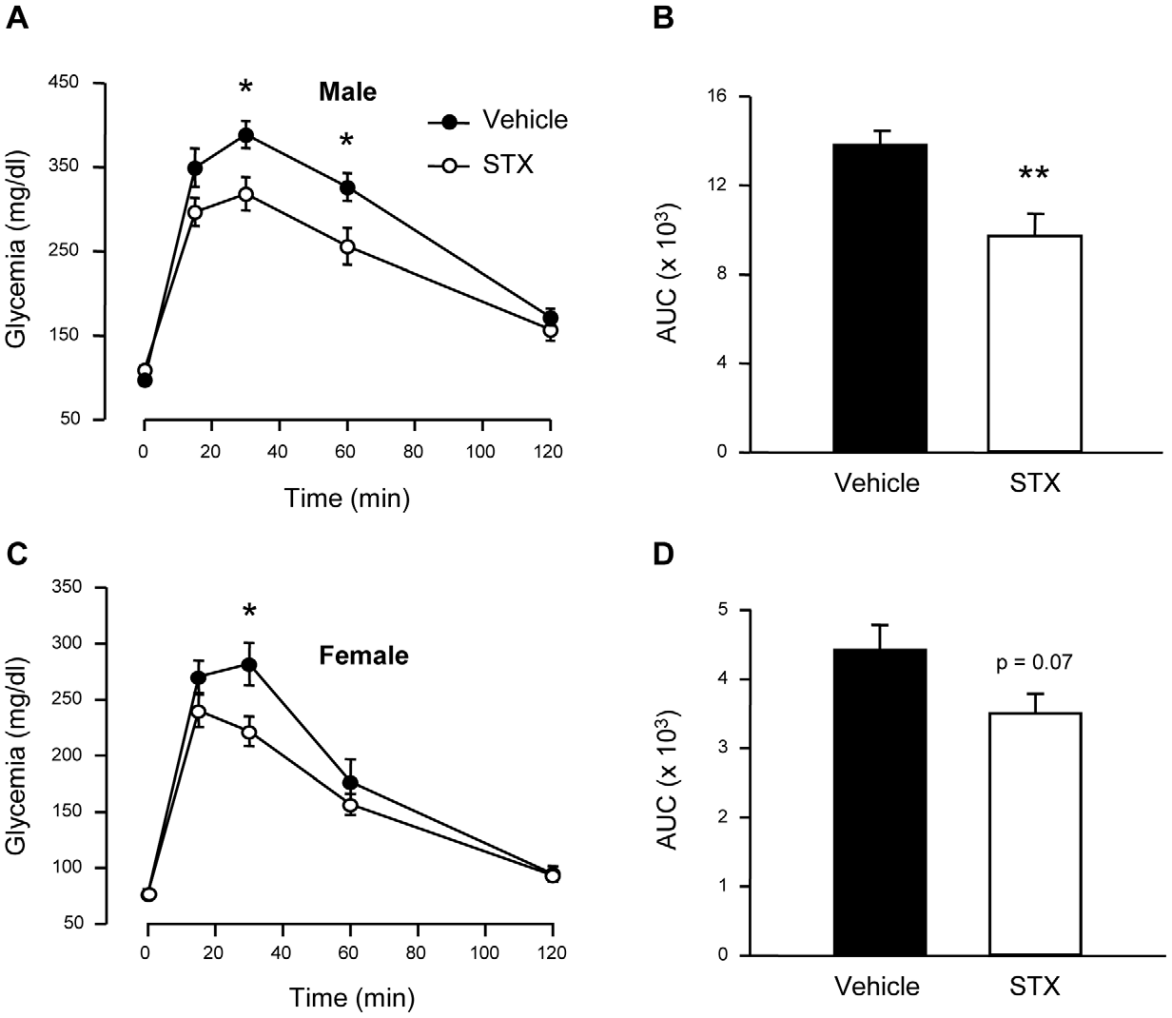
STX improves glucose sensitivity in male mice. A, Intraperitoneal glucose tolerance test (IPGTT) in male mice after injection of Vehicle (•) or 100 µg/kg STX (○). B, Area under the curve in the IPGTT in males; n = 6–7 mice/condition. C, IPGTT in female mice. D, Area under the curve in the IPGTT in females; n = 7 mice/condition. * p<0.05, ** p<0.01.

As mentioned above, pancreatic β-cells are important to maintain blood glucose homeostasis, particularly glucose sensitivity. Given the insulinotropic effect of STX in islets from male mice *ex vivo*, we analyzed the rapid effect of STX on glucose tolerance. For this purpose, overnight fasted male mice were injected with a single i.p. injection of STX (100 µg/kg) in conjunction with a glucose challenge of 2 g/kg (IPGTT). As shown in Figure 4A, glycemia in response to a glucose load after STX administration did not reach the values obtained with vehicle. Glycemia at 30 and 60 min were significantly decreased and the area under the curve (AUC) was also reduced with STX (Fig. 4B). We also performed experiments in female mice, and STX only improved glucose tolerance at 30 min (Fig. 4C), while AUC did not reach statistical significance (p = 0.07) (Fig. 4D). Therefore, we conclude that STX rapidly modulates glucose tolerance in male mice, with only a mild effect in females.

### STX increases plasma insulin in response to a glucose load in male mice

**Figure 5:**
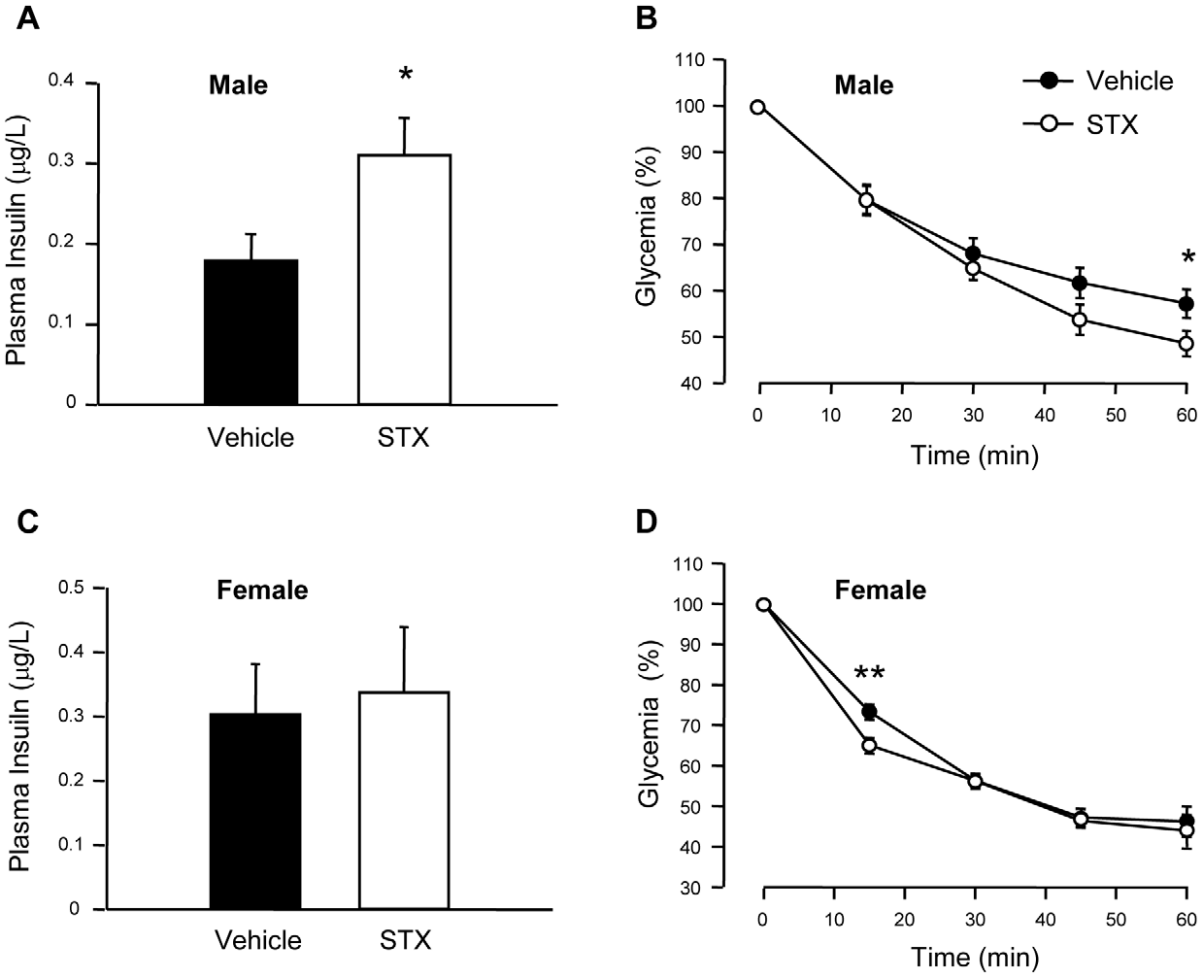
STX increases glucose-induced plasma insulin levels. A, Plasma insulin concentration (µg/l) measured at 30 min after a 2 g/kg glucose load in male mice treated with vehicle or 100 µg/kg STX; n≥7 mice/condition. B, Insulin tolerance test (ITT) in male mice after treatment with vehicle or 100 µg/kg STX; n = 11 mice/condition. C, Plasma insulin concentration (µg/l) measured at 30 min after a 2 g/kg glucose load in female mice treated with vehicle or 100 µg/kg STX; n = 5–6 mice/condition. D, Insulin tolerance test (ITT) in female mice after treatment with vehicle or 100 µg/kg STX; n = 7–8 mice/condition. * p<0.05, ** p<0.01.

Changes in glycemia during an IPGTT are the result of the balance between insulin secretion by the endocrine pancreas and insulin sensitivity in peripheral tissues. To decipher the main component responsible for the increase in glucose tolerance by STX, we measured plasma insulin 30 min after the glucose load and STX administration. In agreement with the insulin secretion experiments, plasma insulin concentration was increased in mice treated with 100 µg STX/kg body weight (Fig. 5A). In addition, we performed an Insulin Tolerance Test (ITT), and we found that STX slightly increased insulin sensitivity, although this effect was mild and statistically significant only at 60 min (Fig. 5B). We also performed these experiments in intact females. As expected, STX did not alter plasma insulin after the glucose load, and insulin resistance was only improved at 15 min (Fig. 5C, D). Therefore, STX rapidly modulates blood glucose homeostasis in male mice mainly by enhancing the plasma insulin response to glucose.

### STX does not alter glucose homeostasis or insulin content after long-term treatment

**Figure 6:**
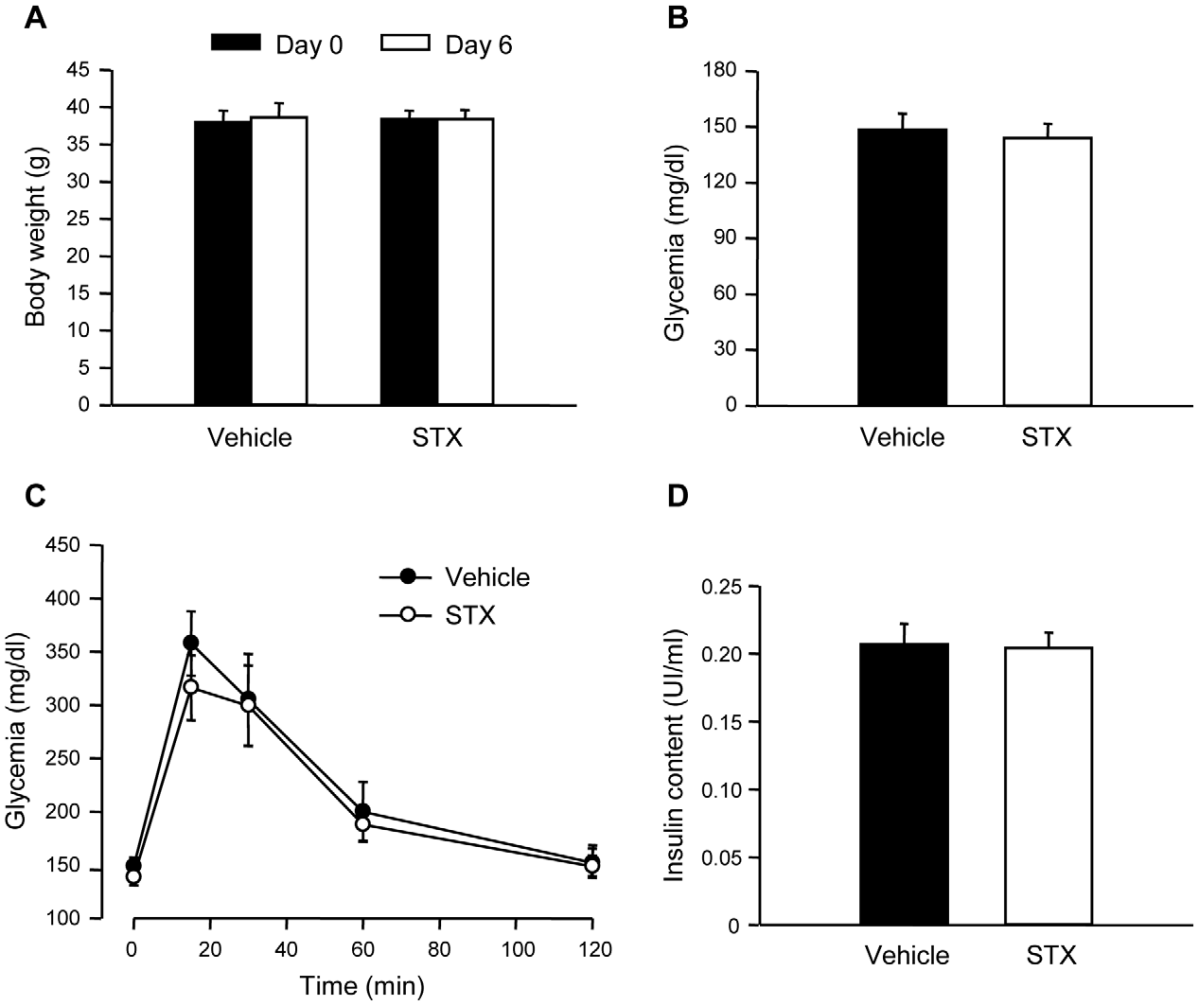
STX does not alter insulin content or blood glucose homeostasis at long-term in male mice. A, Body weight in male mice before and after the 6 day-treatment with either vehicle or STX; n = 5 mice/condition. B, Fasting glycemia in the following morning after the 6-day treatment with vehicle or STX treated mice (n = 5 mice/condition). C, IPGTT in the same mice as in B; vehicle (•), STX (○); n = 4–5 mice/condition. D, Insulin content in isolated islets from vehicle and STX-treated mice; n = 60–120 islets from 2–3 mice/condition.

Our next aim was to study the consequences of a daily dose of STX on blood glucose homeostasis. For this purpose, we injected male mice with a daily dose of 100 µg/Kg body weight for 6 days. This treatment did not alter body weight or fasting glucose levels (Fig. 6A–B). We then performed IPGTT and it was not modified by treatment with STX (Fig. 6C). In addition, insulin content in pancreatic islets was unaltered as shown in Fig. 6D. Therefore, STX at this low dose does not affect body weight, fasting glycemia or IPGTT in male mice or insulin content in pancreatic islets after a 6 day-treatment.

### STX does not alter glucose homeostasis in ovariectomized females

**Figure 7:**
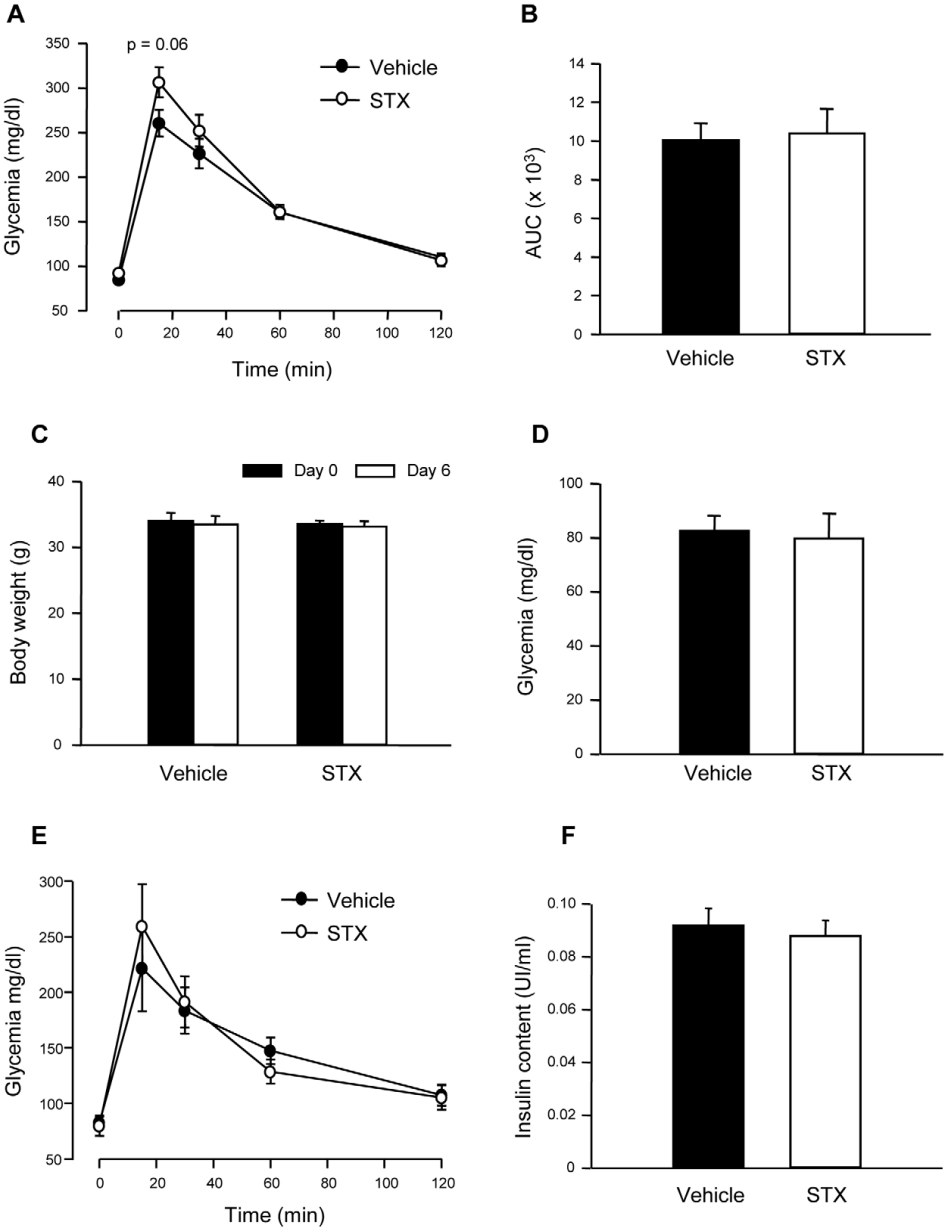
STX does not change glucose homeostasis in ovariectomized female mice. A, Intraperitoneal glucose tolerance test (IPGTT) in ovariectomized female mice after one single dose of vehicle (•) or 100 µg/kg STX (○). B, Area under the curve in the IPGTT in A; n = 7–8 mice/condition. C, Body weight in ovariectomized female mice before and after the 6 day-treatment with either vehicle or STX; n = 5–6 mice/condition. D, Fasting glycemia in the following morning after the 6-day treatment with vehicle or STX treated mice (n = 5–6 mice/condition). E, IPGTT in the same mice as in D; vehicle (•), STX (○); n = 5–6 mice/condition. F, Insulin content in isolated islets from vehicle and STX-treated ovariectomized female mice; n = 110–140 islets from 4–6 mice/condition.

In view of the gender-specific effect of STX in males, we wondered whether this was due to the presence of 17β-estradiol in the females. Therefore, we studied the effect of STX in ovariectomized (OVX) females. The experiments were performed 14 days after ovariectomy. As in intact females, a single dose of 100 µg/Kg STX failed to alter blood glucose homeostasis as shown in figures 7A–B. We then treated OVX females for 6 days with a daily dose of 100 µg/Kg STX. As in males, the compound did not alter body weight (Fig. 7C). In addition, fasting glycemia and IPGTT performed on the morning of the seventh day were not modified by STX (Fig. 7D–E). Insulin content from isolated islets was indifferent to the 6-day treatment with 100 µg/Kg STX (Fig. 7F). Therefore, STX does not alter blood glucose homeostasis in ovariectomized females, either after an acute or a long-term treatment with a 100 µg/Kg dose of STX.

### STX does not improve glucose homeostasis in a mice model of mild diabetes

**Figure 8:**
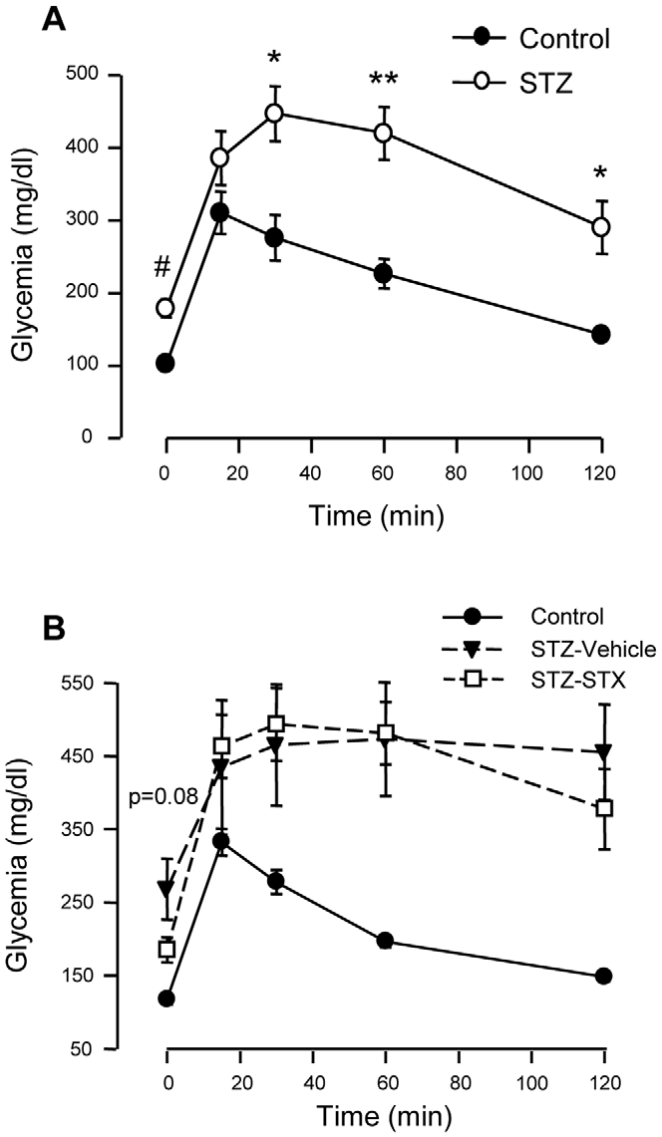
Long-term treatment with STX does not improve glucose homeostasis in a model of mild diabetes. A, IPGTT in control mice or treated with nicotinamide and streptozotocin (STZ) to generate glucose intolerance. B, IPGTT in the same mice after 6 days treatment with either vehicle (Control •; STZ-Vehicle ▾) or 100 µg/Kg STX (STZ-STX ○); n = 5–8 mice/condition; * p<0.005; ** p<0.001; # p<0.0005; p = 0.08 STZ-STX vs. STZ-Vehicle at 0 min (fasting glycemia).

Given the insulinotropic effect of STX *ex vivo* and the improvement of glucose tolerance *in vivo*, we decided to study the importance of these effects in a situation of β-cell dysfunction. For this purpose, we used a model of mild diabetes induced with nicotinamide and streptozotocin (STZ). Ten days after this treatment, surviving mice were tested for glucose intolerance (Fig. 8A). Animals treated with STZ were then divided in two groups, one receiving a daily i.p. injection of vehicle, the other 100 µg/Kg STX, for 6 days. As shown in Figure 8B, the 6-day treatment with this dose of STX reduced hyperglycemia, although not significantly (p = 0.08), while treatment did not improve glucose tolerance in mice tested on the morning of the seventh day.

## Discussion

In this work we show that low concentrations of STX rapidly enhanced glucose-induced insulin secretion in islets, increased calcium response to glucose and closed the K_ATP_ channels in pancreatic β-cells. That the effect of STX is direct on β-cells is indicated by the calcium and patch-clamp experiments performed in isolated cells. The insulinotropic effect of STX was only produced in islets from male mice, while no effect was obtained in islets from female mice. In addition, the anti-estrogen ICI 182,780 blocked the insulinotropic effect of STX. *In vivo*, STX improved glucose tolerance and enhanced glucose-induced plasma insulin levels in male mice, with only a slight increase in insulin sensitivity. In females, the effect on blood glucose homeostasis was quite modest and no change was observed in plasma insulin in response to the glucose load or insulin sensitivity. After a 6 day-treatment with a low dose, STX did not modify body weight, glucose tolerance or islet insulin content. STX was also ineffective in modifying glucose homeostasis in ovariectomized females either after an acute dose or a long-term treatment. It did not improve glucose homeostasis in male mice from a model of glucose intolerance after 6 days of treatment.

The data presented here suggest that STX has a gender-specific effect in modulating pancreatic β-cell function. This insulinotropic effect of STX in pancreatic β-cells from male mice may be mediated by the closure of K_ATP_ channels and the increase in intracellular calcium concentration observed. The *in vivo* improvement in glucose tolerance in males after an acute dose appears to be mostly due to the enhanced insulin secretion in β-cells.

Physiological concentrations of the endogenous hormone 17β-estradiol have been reported to increase insulin secretion in pancreatic islets of Langerhans. This effect is mediated by the modulation of the K_ATP_ channel activity and calcium signaling [2], [40]. In addition, 17β-estradiol attenuates the glycemic response to fatty acids *in vivo* [41]. The results presented here show that similar doses of STX mimic the effects of 17β-estradiol on insulin secretion *ex vivo* in islets from male mice. In addition, STX also improves glucose tolerance *in vivo* in response to a glucose load. However, differences exist between the actions of 17β-estradiol and STX. On one hand, 17β-estradiol has proven to be effective in islets from both males and females [19], [36], while STX is only effective in males. On the other hand, 17β-estradiol increases insulin content and produces mild glucose intolerance after 4 days of treatment [41], while STX does not alter these parameters after a 6-day treatment. In addition, the insulinotropic effect of 17β-estradiol is mediated by ERβ, while STX does not bind to this receptor. In spite of this, the effect of STX in pancreatic β-cells, as in hypothalamic neurons [27], [30], is blocked by the selective estrogen receptor antagonist ICI 182,780, suggesting a novel estrogen receptor involved.

Blood glucose homeostasis is maintained by the interplay of several tissues: liver, brain, adipose tissue, skeletal muscle and the endocrine pancreas. Pancreatic β-cells are key regulators of glucose tolerance and proper β-cell function is essential to maintain blood glucose homeostasis [1]. Within the brain, the hypothalamus is involved in glucose and energy homeostasis [42]. Indeed, insulin directly infused into the arcuate nucleus of the hypothalamus (ARC) reduces hepatic glucose production, an effect that is inhibited with K_ATP_ blockers [43]. Activation of hypothalamic K_ATP_ channels lowers plasma glucose through inhibition of hepatic gluconeogenesis [44]. STX has been shown to modulate the activity of hypothalamic neurons, particularly dopamine and POMC neurons, as well as the activity of K_ATP_ channels in GnRH neurons [25], [27], [28], [30]. STX, along with 17β-estradiol, prevents weight gain after ovariectomy in guinea pigs, probably due, in part, to a decrease in food intake [27], [29], [45]. Therefore, all these findings suggest that STX may have a broader impact on blood glucose homeostasis not only through its effects on pancreatic β-cell function, but also through the modulation of hypothalamic neurons controlling energy homeostasis [27], [29].

Data presented here show that 100 µg/Kg STX improves glucose homeostasis in male mice after one dose administered at the same time as a glucose load. However, no effect is observed 24 hours after the cessation of a 6-day treatment with the same dose, either in normal mice or in a model of mild diabetes. The reason for these differences may be the pharmacokinetics of the compound, which have not been extensively studied. In fact, the pharmacokinetic properties determine the administration frequency of insulinotropic anti-diabetic drugs such as sulphonylureas and glinides, both K_ATP_ channel blockers [46], [47]. Glinides, for example need to be administered more frequently during the day than sulphonylureas due to their shorter circulating half-life [47]. In addition, the precise time of the administration may change the glucose-lowering effects of these drugs, as in the case of tolbutamide [48]. Indeed, in guinea pigs, the least observable effective concentration (LOEC) of STX for modulation of energy homeostasis is 2 mg/kg [27].

In this work we show the gender-specific effect of STX in mice. While 100 µg/Kg STX improves glucose homeostasis in male mice, females are insensitive even after ovariectomy. The lack of effect with STX after ovariectomy suggests that the differences are not due to circulating estrogens, but rather to developmental differences that are not reversed post-ovariectomy, similar to the loss of the leptin receptor in POMC neurons [49]. Gender-specific differences are not uncommon in metabolism. There is evidence that insulin sensitivity differs between males and females [50], [51]. Moreover, many studies have shown that women have lower diabetes prevalence than men [11], [52]–[54]. In diverse animal models of glucose intolerance, insulin resistance and diabetes, males show a stronger phenotype than females [55], [56]. Therefore, it is not surprising that the response to drugs aimed at metabolic targets depend on the sex, as it is in the case with STX. In fact, men and women respond differently to some medications and therapeutics [57].

In summary the work presented here is the first approach to a full characterization of STX as a potential anti-diabetic agent. Since STX has no uterotropic effects and does not bind to ERα or ERβ, it may be a novel SERM with the beneficial effects of estrogen therapy without many of the deleterious side effects.
