# Correction: Insulinotropic Effect of the Non-Steroidal Compound STX in Pancreatic β-Cells

**DOI:** 10.1371/annotation/c1da4989-c6e8-43c2-9a8b-1462af83ae5e

**Published:** 2012-05-30

**Authors:** Ana B. Ropero, Paloma Alonso-Magdalena, Sergi Soriano, Pablo Juan-Picó, Troy A. Roepke, Martin J. Kelly, Ángel Nadal

There were errors in the Funding section. The correct funding information is as follows: This work was supported by Ministerio de Ciencia e Innovación grants BFU2008-01492 and BFU2011-28358, by the Generalitat Valenciana grants GV/2009/056 and Prometeo/2011/080, by a UMH-Bancaja grant and by the US Public Health Service grant DK 68098. CIBERDEM is an initiative of Instituto de Salud Carlos III. The funders had no role in study design, data collection and analysis, decision to publish, or preparation of the manuscript.

